# Control of plasmonic nanoantennas by reversible metal-insulator transition

**DOI:** 10.1038/srep13997

**Published:** 2015-09-11

**Authors:** Yohannes Abate, Robert E. Marvel, Jed I. Ziegler, Sampath Gamage, Mohammad H. Javani, Mark I. Stockman, Richard F. Haglund

**Affiliations:** 1 Center for Nano-Optics (CeNO), Georgia State University, Atlanta, Georgia 30303, USA; 2 Department of Physics and Astronomy, Georgia State University, Atlanta, Georgia 30303, USA; 3 Interdisciplinary Materials Science Program, Vanderbilt University, Nashville, TN 37235-1406; 4 Department of Physics and Astronomy, Vanderbilt University, Nashville, TN 37235-1807.

## Abstract

We demonstrate dynamic reversible switching of VO_2_ insulator-to-metal transition (IMT) locally on the scale of 15 nm or less and control of nanoantennas, observed for the first time in the near-field. Using polarization-selective near-field imaging techniques, we simultaneously monitor the IMT in VO_2_ and the change of plasmons on gold infrared nanoantennas. Structured nanodomains of the metallic VO_2_ locally and reversibly transform infrared plasmonic dipole nanoantennas to monopole nanoantennas. Fundamentally, the IMT in VO_2_ can be triggered on femtosecond timescale to allow ultrafast nanoscale control of optical phenomena. These unique features open up promising novel applications in active nanophotonics.

Realizing the potential of nanophotonics for signal and information processing requires control and manipulation of light at subwavelength scales. Optical energy concentration on the nanoscale is achieved on metal nanostructures due to polar electronic modes called surface plasmons (SPs)^1^,^2^,^3^. Photonic crystals are used for complete reflection, guiding, and confinement of light^4^, while metamaterials are used to transform light in unconventional ways, making possible such novel devices as perfect absorbers^5^ , circular polarizers^6^, and selectively reflecting surfaces^7^. While novel devices that control the propagation of light on the nanoscale have been demonstrated^1^,^8^,^9^,^10^, *active* nanoscale control of optical physics in nanostructures is still a major challenge in^11^,^12^,^13^, and a bottleneck for, related technologies. Nanoscopic control of the insulator and metallic phases of vanadium dioxide (VO_2_) would open up a universe of applications in nanophotonics via modulation of the local dielectric environment of nanophotonic structures, allowing them to function as active devices.

In this Article, we demonstrate unprecedented active nanoscale control of concentration of light by single plasmonic infrared antennas in the near-field. The active control of the dielectric environment by the insulator-to-metal transition (IMT) in vanadium oxide (VO_2_), dynamically the transforms nanoantennas from dipole to monopole and back. We utilize the local, reversible change of refractive index of VO_2_ that undergoes a first-order phase transition from an insulating monoclinic phase to a metallic rutile phase near 70 °C in bulk single crystals^14^, the transition can also be induced by strain^15^,^16^ and ultrafast light pulses^17^,^18^. In polycrystalline VO_2_ thin films, the IMT begins as conductive nanodomains nucleate and with increasing temperature evolve to interconnect in a percolative fashion throughout the film^19^. At intermediate stages of the IMT, insulating and metallic phases coexist, forming a network of high- and low-conductivity nanodomains throughout the film. Since the metallic and insulating nanodomains have substantially different refractive indices, VO_2_ films provide for direct local control of the dielectric environment at nanometer spatial dimensions, which, in turn, can directly modulate optical responses of nanophotonic structures.

So far, the effects of the VO_2_ IMT on plasmonic nanostructures have been studied only in the far-field^20^, so that understanding and active control of the near-field interaction by the VO_2_ domains has been elusive. Here, we present an experimental study of nanoscale interactions of plasmonic structures with VO_2_ undergoing the IMT using scattering-scanning near-field optical microscopy (s-SNOM)^21^, which images local vector near-fields with minimal perturbation, indispensable for the study of nanoplasmonic phenomena^22^,^23^,^24^,^25^. Image formation in s-SNOM relies on the effective polarizability of tip-sample complex, allowing image contrast that is based on local dielectric environment, which is ideal for nanoscale imaging of IMT.

## Results

**Figure 1:**
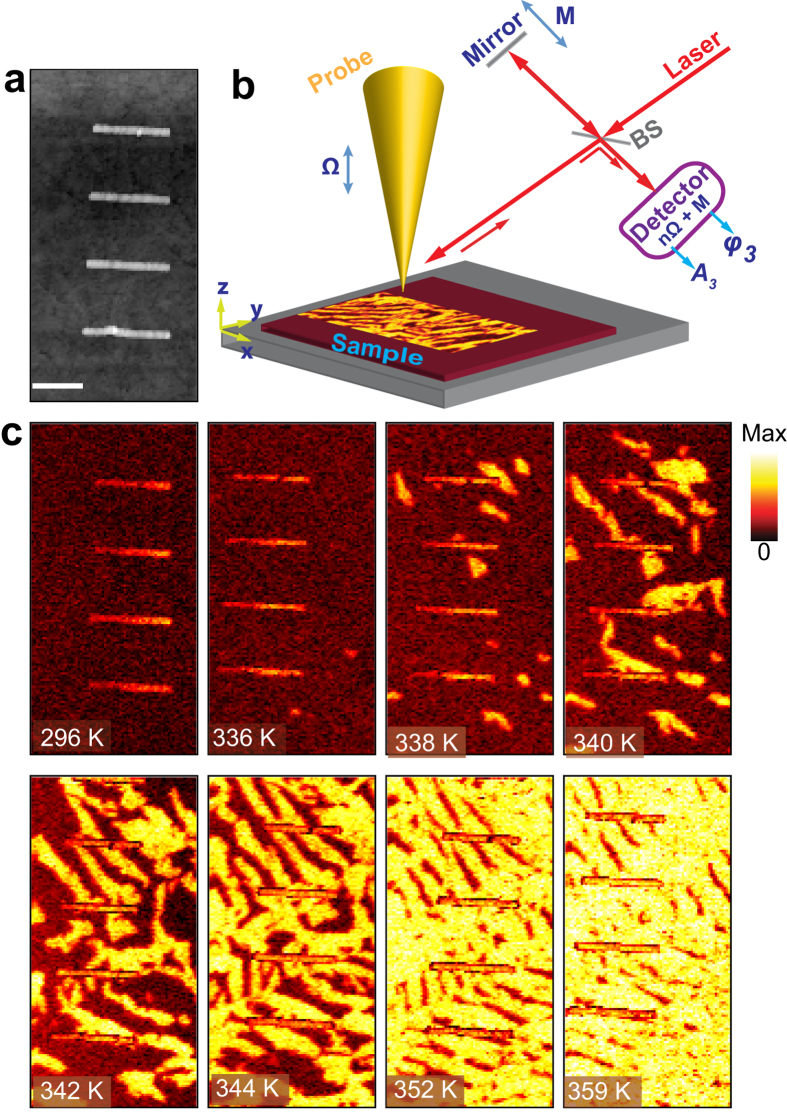
Temperature dependent nanoscale near-field amplitude images of IMT emergence and progress around the infrared antennas. (**a**) Topography of the four antennas on VO_2_ film. The scale bar indicates 1 μm. (**b**) Schematic of the s-SNOM experimental setup, which allows polarization-controlled simultaneous imaging of IR plasmonic antenna modes together with the phase spatial evolution of VO_2_ phase transition in amplitude and phase. The coordinate system is positioned so that the *y* axis is directed along nanorods and the *z*-axis is normal to the plane of the nanostructure. (**c**) Temperature-dependent near-field amplitude images reveal IMT via forming initial metallic nanodomains, which grow and connect to stripes and quasi-uniform metallic phase.

The model system comprised an array of identical infrared plasmonic nanoantennas: gold nanorods fabricated by e-beam lithography on a 100 nm VO_2_ film grown on a [100]_R_ Si substrate (Fig. 1a). The dimensions of the rods (~2510 nm × 232 nm × 30 nm) were selected to be near-field resonant at mid-infrared frequencies (10.7 μm vacuum wavelength). Near-field optical images were acquired using a commercial s-SNOM system (neaspec.com). A linearly polarized CO_2_ laser is focused on the tip–sample interface at an angle of 45^°^ to the sample surface (Fig. 1b). The scattered field is detected by phase-modulation (pseudo-heterodyne) interferometry, yielding topography, amplitude, and phase images^23^,^24^,^25^,^26^.

First, we investigate the temperature-dependent emergence of metallic nanodomains during the IMT. The temperature was controlled by a heater, and a p-polarized (in the *yz* plane)) excitation laser was tuned to 10.7 μm, with the p-polarized detection (Fig. 1 and Methods). Figure 1 shows the third harmonic (of the tip oscillation frequency) amplitude (A_3_) images of VO_2_ and the Au nanorod array as a function of temperature. These images reveal the emergence of an anisotropic network of bright domains as the IMT progresses with increasing temperature. While the onset of these local metallic domains appears random, the stripes in fact evolve by connecting to the existing domains in crystallographically preferred directions until a quasi-uniform rutile phase emerges at high temperatures. In the s-SNOM, higher local polarizability in the sample results in stronger near-field optical contrast; hence these near-field images faithfully represent temperature-driven formation of metallic domains in the VO_2_ film^19^.

The formation of stripe phases was noted soon after the discovery of VO_2_. Subsequently, a link was inferred between the stripe phase and substrate strain in high-quality single crystals and thick (250 nm) epitaxial grown by ion-beam assisted sputtering on TiO_2_ [100]_R_ surfaces^27^,^28^. Stripe formation is also correlated with the power of a near-IR pump laser (1.56 μm), close to the surface-plasmon resonance of the VO_2_.

**Figure 2:**
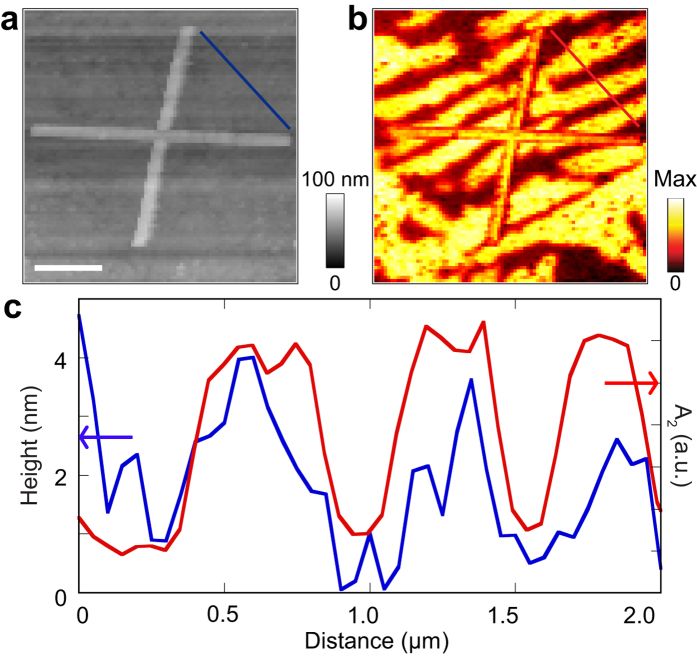
Topography correlation with near-field signal. (**a**) Topography and (**b**) third harmonic near-field amplitude image showing four Au infrared antennas on VO_2_ film. (**c**) topography line profile superimposed on amplitude line profile at the marked positions shown by the lines shown in (**a,b**).

Here, the VO_2_ films are thinner (100 nm), polycrystalline, and have no epitaxial relationship to the Si substrate. Nevertheless, the stripe phase appears during the transition from monoclinic to rutile, suggesting that it may occur simply because of localized, in-plane (*xy*) strains that develop at the film surface as individual grains of VO_2_ begin to change phase, without any reference to the substrate. This possibility is further supported by the very small height of the stripes seen in the present experiment (Fig. 2).

The IMT metallic stripes appear with localized near-uniform spacing in the near-field amplitude image at an intermediate phase coexistence temperature. These are accompanied by correlated topographic modulation as clearly shown in Fig. 2. Topography (Fig. 2a) and near-field amplitude (Fig. 2b) images are taken at excitation laser wavelength, λ = 10.7 μm and temperature, T = 344 K, along with the line profile sketches (Fig. 2c). The topographic variation in our case is smaller (0–3 nm) and the correlation weaker compared to what was observed on a TiO_2_ substrate (0–5 nm)^27^. The correlation of the topographic line profile with the periodic stripes is due to a structural change in VO_2_ during MIT, which results from the modulation of the rutile and the monoclinic axes.

**Figure 3:**
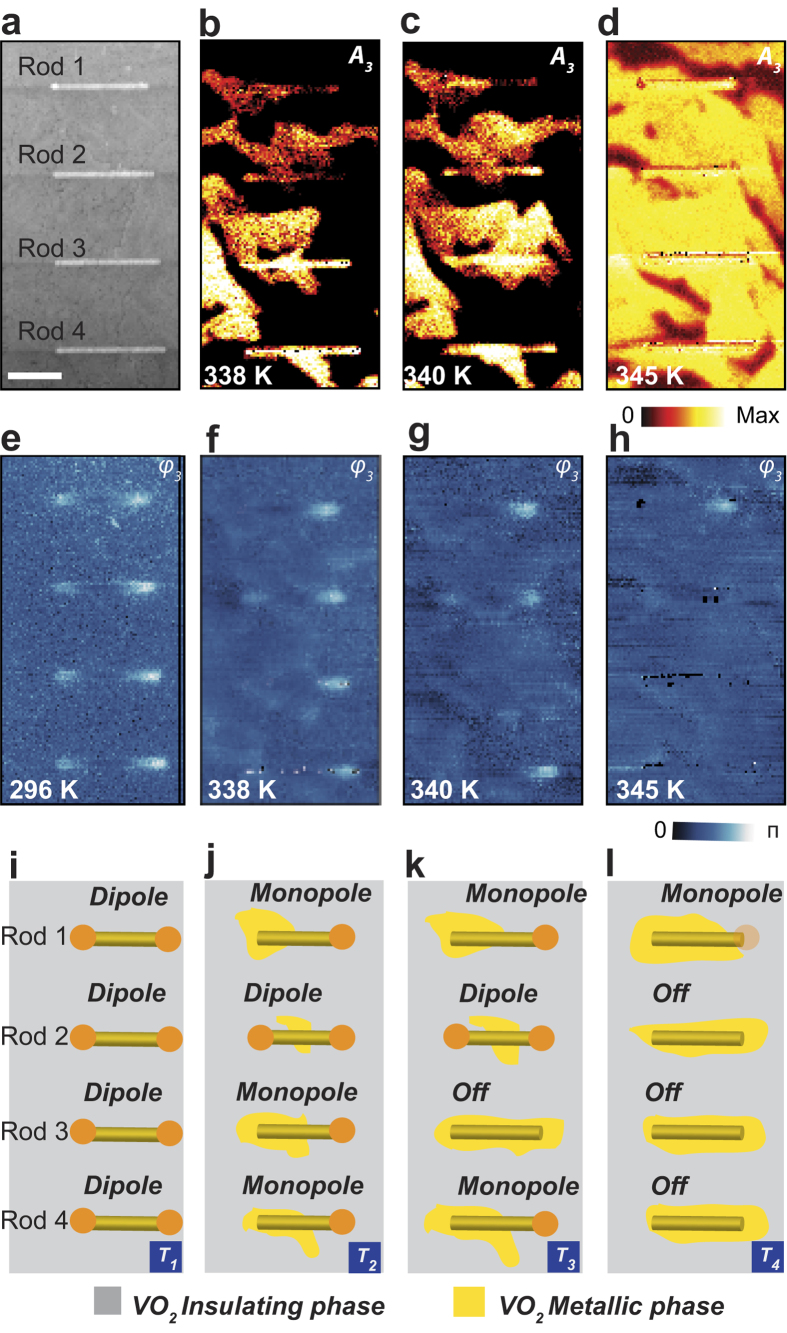
Temperature-controlled IMT and antenna near-field images. Near-field 3^rd^ harmonic amplitude (**b–d**) and phase (**e–h**) images. Schematics (**i–l**) describing experimental results of IR plasmonic antenna modes simultaneously with VO_2_ thin film IMT domain formation and propagation.

To directly visualize plasmonic modes of the antennas and their interaction with the IMT of the VO_2_ film, we implement in-plane polarization-selective excitation (s-excitation, i.e. polarized along the *y* direction) and in-plane detection (s-detection), which is referred to s/s imaging. Figure 3 shows topography, third-harmonic s/s near-field amplitudes, and phase images of the antennas on the VO_2_ film for different temperatures. The four IR antennas are nominally identical (Fig. 3a), making it possible to compare the effects of VO_2_ IMT on them. The amplitude images (Fig. 3b–d) show bright and dark optical contrast due to the coexisting insulating (dark) and metallic (bright) phases affecting the nanoscale dielectric environment of the antennas. The metallic phase begins to form randomly with increasing temperature. As a result, portions of the antennas are located partly on the metallic and partly on the insulating phases of VO_2_ as observed in the amplitude images (Fig. 3b–d). The amplitude images allow one to see the change of nanoscale field magnitudes and the metallic phase formation. At the same time, the near-field *phase* images Fig. 3e–h) are less sensitive to material contrast but allow one to follow the dipolar mode modification on each of the four antennas due to IMT. They display strong phase contrast at the rod ends. The VO_2_ regions exhibit very weak phase contrast as shown in Fig. 3e–h, which is independent of excitation or detection polarizations.

**Figure 4:**
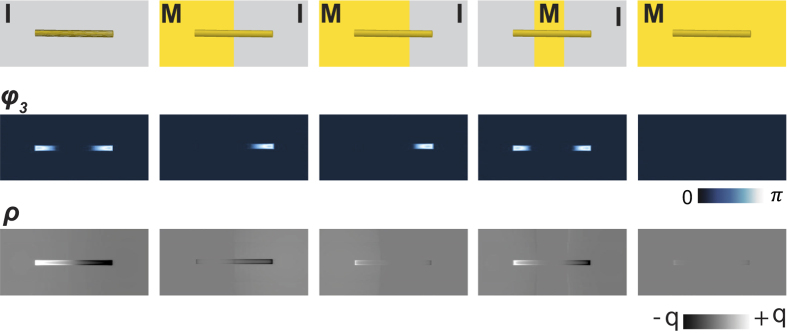
Finite difference time-domain simulations. Single antenna field intensity, phase and surface charge images of FDTD simulations. On schematic of the upper panel, I and M denote insulator and metal phases, respectively.

At room temperature, all antennas display identically the expected pronounced dipolar phase contrast at their ends, as shown in Fig. 3e. At higher temperatures, all antennas whose one end is situated on the metallic phase turn from dipole to monopole as evident for Rod 1 (Fig. 3f–h), Rod 3 (Fig. 3f), and Rod 4 (Fig. 3f–g). At even higher temperature, when the amplitude image shows that most of the film is in metallic phase (Fig. 3d), both dipole and monopole antenna modes of Rods 2, 3 and 4 turn off (Fig. 3h) completely. An interesting case is Rod 2: despite the middle part of the rod sitting on the metallic phase (Fig. 3c), it still retains its dipole characteristics (Fig. 3g) since both ends are on the insulating grains. It only turns off at higher temperature when the entire antenna is situated on metal (Fig. 3h). These results are interpreted in schematics shown in Fig. 3i–l. This interpretation is supported by numerical calculations performed using the finite difference time-domain (FDTD) simulations (Lumerical Inc.,) shown in Fig. 4, which are in excellent qualitative agreement with experiment.

**Figure 5:**
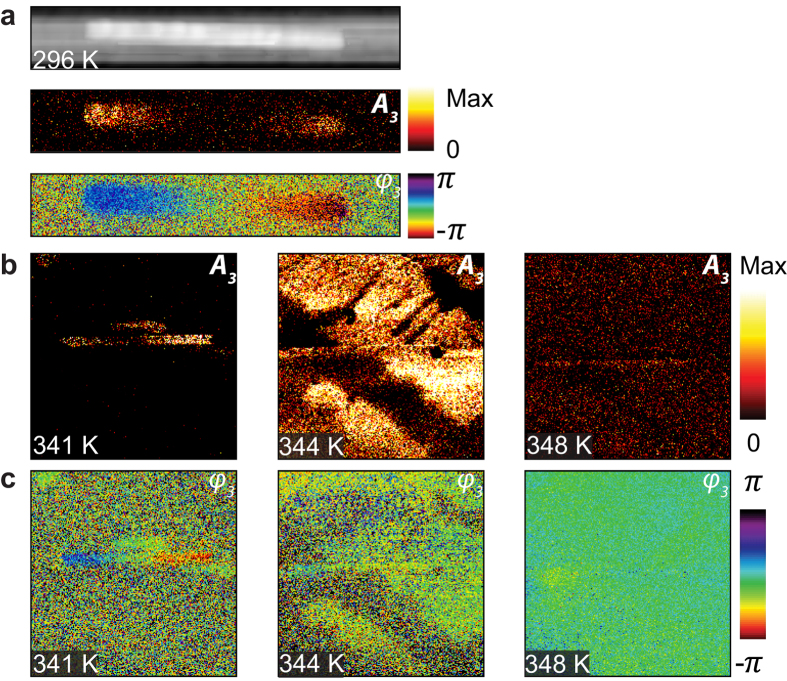
Cross-polarized s/p excitation-detection imaging of plasmons and IMT of VO_2_. (**a**) Topography, near-field amplitude and phase images of IR antenna at room temperature. (**b**) near-field amplitude and (**c**) near-field phase images of antenna on VO_2_ film at three different temperatures.

Further tracking of active dipole-to-monopole transformation of plasmonic antennas can be performed using s-excitation and p-detection (s/p) cross-polarization selective imaging see Fig. 5. Panel a displays topography, third harmonic optical near-field amplitude and phase images of an Au antenna on the VO_2_ substrate at room temperature. The amplitude image displays a stronger optical contrast at rod ends and the phase image shows π phase difference between the rod ends. The amplitude and phase images both exhibit the signature of a dipolar mode of a plasmonic rod expected from S/P cross-polarized excitation/detection experimental method.

Figure 5b shows amplitude contrast of the Au dipolar mode simultaneously with the metallic domain at the onset of phase transition at T = 341 K. Here, the amplitude optical contrast at rod ends is masked by the bright metallic domain contrast of the VO_2_ film, and is not clearly distinguishable in the amplitude image. In contrast, the phase image (Fig. 5c) distinctly discriminates the Au plasmonic rod from the metallic background of VO_2_ film. As temperature increases (T = 344 K), the metallic phase grows and a portion of one side of the rod sits on the metal and the other side sits on the insulator. The phase image between the rod ends indicates dipole (at T = 296 K and 341 K) to monopole (at T = 344 K) to off (at T = 348 K) transformation of the nanoantenna.

In summary, we have shown the first experimental evidence that *near-field* local optical processes in plasmonic nanostructures can be directly and actively controlled by nanodomains in VO_2_ film as it undergoes the IMT. Depending on the precise location of the nanoantennas with respect to metallic and insulating domains in the VO_2_ film on the scale of 15 nm or less, the IMT reversibly transforms infrared plasmonic dipole antennas to monopole antennas or switches them off. We envision that such dynamic active control of the nanoscale interaction of light with nanostructured materials, which can potentially be ultrafast, will open up diverse applications in nanooptics and the related technologies.

## Methods

### Near-field microscopy

The microscope is a commercial s-SNOM system (neaspec.com), which has been described in detail elsewhere. A probing s or p linearly polarized CO_2_ laser is focused on the tip–sample interface at an angle of 45^°^ from the sample surface. The scattered field is acquired using a phase modulation, or pseudoheterodyne interferometry. The background signal is suppressed by vertical tip oscillation at the mechanical resonance frequency of the cantilever (*f*_*0*_ ~ 285 kHz) and demodulation of the detector signal at higher harmonics *nf*_*0*_ (commonly *n* = 2, 3) of the tip resonance frequency. The combined scattered field from the tip and the reference beam pass through a linear polarizer which further selects the p/s polarization of the measured signal for analysis.

### Sample fabrication

Amorphous vanadium dioxide films nominally 100 nm thick were deposited on a silicon (100) substrates by electron beam evaporation of a V_2_O_4_ powder precursor^29^. Annealing at 45 °C in 250 mTorr oxygen crystallized the amorphous films into switching VO_2_ with 67 °C phase-transition temperature. A poly(methyl methacrylate) resist (PMMA 495 A4 from Microchem) was spun onto the VO_2_ films before fabricating the antenna arrays by electron beam lithography with a Raith eLINE system. After development of the resist, 50 nm of gold was deposited by thermal evaporation; the remaining resist was removed by lift-off in warm acetone.

### Numerical calculations

Experimental results are theoretically interpreted with the aid of finite difference time-domain (FDTD) simulations (Lumerical Inc., lumerical.com). For all simulations each Au rod has dimensions l = 2512 nm, w = 232 nm and h = 30 nm. These dimensions were averaged from topography scan measurements. Each particle is simulated atop a VO_2_ substrate. The optical excitation source used for the simulation is a mid infrared (10.7 μm) plane wave. The simulation is performed by assuming a uniform VO_2_ film with complex dielectric constant ε = 4.9 for the monoclinic insulating phase and ε = −35 + 119i for the rutile metallic phase at λ = 10.7 m^30^. The simulation is performed using both s/s and s/p polarization selective excitation-detection methods.

## Additional Information

**How to cite this article**: Abate, Y. *et al.* Control of plasmonic nanoantennas by reversible metal-insulator transition. *Sci. Rep.* **5**, 13997; doi: 10.1038/srep13997 (2015).
